# Quantitative CT analysis of honeycombing area predicts mortality in idiopathic pulmonary fibrosis with definite usual interstitial pneumonia pattern: A retrospective cohort study

**DOI:** 10.1371/journal.pone.0214278

**Published:** 2019-03-21

**Authors:** Hiroaki Nakagawa, Emiko Ogawa, Kentaro Fukunaga, Daisuke Kinose, Masafumi Yamaguchi, Taishi Nagao, Sachiko Tanaka-Mizuno, Yasutaka Nakano

**Affiliations:** 1 Division of Respiratory Medicine, Department of Internal Medicine, Shiga University of Medical Science, Shiga, Japan; 2 Health Administration Center, Shiga University of Medical Science, Shiga, Japan; 3 Department of Medical Statistics, Shiga University of Medical Science, Shiga, Japan; Helmholtz-Zentrum Munich, GERMANY

## Abstract

**Background:**

Honeycombing on high-resolution computed tomography (HRCT) images is a key finding in idiopathic pulmonary fibrosis (IPF). In IPF, honeycombing area determined by quantitative CT analysis is correlated with pulmonary function test findings. We hypothesized that quantitative CT-derived honeycombing area (HA) might predict mortality in patients with IPF.

**Materials and methods:**

Chest HRCT images of 52 IPF patients with definite usual interstitial pneumonia (UIP) pattern were retrospectively evaluated. Mortality data up to July 31, 2016, were recorded. Using a computer-aided system, HA and percentage of HA (%HA) were measured quantitatively. Predictors of 3-year mortality were evaluated using logistic regression models.

**Results:**

The median %HA, %predicted forced vital capacity (FVC) and composite physiologic index (CPI) were 3.8%, 83.6%, and 33.6, respectively. According to GAP (gender, age, and physiology) stage, 20, 14, and 5 patients were classified under stages I-II-III, respectively. Percentage of HA was significantly correlated with %FVC, CPI, and GAP stage (all, *p* < 0.001). In univariate analysis, %HA, %FVC, and CPI were statistically significant predictors of mortality. In multivariate analysis using the stepwise regression method, only %HA (odds ratio [OR], 1.27; *p* = 0.011) was a significant independent predictors of mortality. Patients with %HA ≥ 4.8% had significantly lower survival rates than those with lesser %HA (median survival time, 1.3 vs 5.0 years; log-rank test; *p* < 0.001).

**Conclusion:**

Quantitative CT-derived HA might be an important and independent predictor of mortality in IPF patients with definite UIP pattern.

## Introduction

Idiopathic pulmonary fibrosis (IPF) is a specific form of chronic and progressive fibrosing interstitial pneumonia of unknown cause [1–3]. Honeycombing is one of the key findings on high-resolution computed tomography (HRCT) images in IPF with definite usual interstitial pneumonia (UIP) pattern. The extent of honeycombing area on HRCT images is an independent predictor of mortality in IPF [1, 4, 5]. Therefore, quantifying the extent of honeycombing on HRCT images allows better management of patients with IPF. Experts have evaluated the extent of honeycombing using CT visual scores [6–9]. However, this evaluation process is not uniform, yielding only fair to moderate agreement regarding honeycombing area among expert radiologists and pulmonologists [10]. The 2011 international statement also mentions the lack of uniformity in approach towards quantification of honeycombing area [4]. Recently, there are some reports about computer-based quantitative CT analyses [11–18]. These methods may be the useful and reliable for objective assessment of HRCT findings including honeycombing. However, the algorithms of their methods remain unclear, and not everyone can use these methods.

We recently developed a computer-aided system for quantitative CT analysis of honeycombing area in IPF [19]. This system has no interobserver errors and could, therefore, address the issues associated with current quantification methods for honeycombing area. Furthermore, everyone can use this system through the public-domain computer program, ImageJ.

Pulmonary function tests (PFTs) are a standard approach for objective monitoring of patients with IPF. Both forced vital capacity (FVC) and diffusing capacity of the lungs for carbon monoxide (DL_CO_) are strongly correlated with disease extent determined histologically or estimated visually using CT images [1]. However, in patients with IPF, interpretation of PFT findings is confounded by coexisting emphysema [20, 21]. Therefore, the composite physiologic index (CPI) was developed to improve on previous prognostic measures in IPF by adjusting for emphysema [20]. There exists another composite index, the GAP stage, which uses gender (G), age (A), and physiology (P) (FVC and DL_CO_) variables for predicting mortality in IPF [22]. Although PFTs are very important in both these indexes, one should be aware that patients with severe IPF may not be able to perform PFTs.

The aim of this study was to assess the relationship between honeycombing area determined by quantitative CT analysis and mortality in patients with IPF. We hypothesized that quantitative CT-derived honeycombing area (HA) might predict survival in IPF with definite UIP pattern.

## Materials and methods

### Study design and patients

All IPF patients with definite UIP pattern—diagnosed in accordance with the 2011 IPF guidelines—who visited the outpatient clinic of our hospital between April 2011 and March 2014 were enrolled in this study. Patients with secondary interstitial pneumonitis (such as collagen vascular disease, pneumonia caused by occupational or environmental exposure, chronic hypersensitivity pneumonitis, and drug-induced pneumonia) were excluded. Mortality and survival data up to July 31, 2016, were recorded. In order to evaluate 3-year mortality, patients with follow-up period < 3 years were excluded. For comparison of clinical features, patients with IPF were classified as “alive” or “deceased” on the basis of survival status at the end of the 3-year follow-up period. The study protocol conformed to the Declaration of Helsinki and was approved by the ethics committee of Shiga University of Medical Science (approval number 24–182; May 1, 2013), with a waiver of the need for informed consent because of the retrospective study design.

Chest HRCT images at the first visit were acquired using Toshiba Aquillion ONE (Toshiba Medical Systems Corp., Otawara, Tochigi, Japan) or SOMATOM Sensation Cardiac (Siemens Japan K.K., Shinagawa, Tokyo, Japan). The technical parameter for Toshiba Aquillion ONE was as follows: slice thickness 0.5–1.0 mm, x-ray voltage 120kVp, x-ray median current 200mA (range 80-350mA), rotation speed 0.5 sec, and reconstruction algorithm FC52. The technical parameter for SOMATOM Sensation Cardiac: slice thickness 0.5–1.0 mm, x-ray voltage 120kVp, x-ray median current 288mA (range 226-481mA), rotation speed 0.375 sec, and reconstruction algorithm B70f. The images used for the analysis were selected with 10-mm intervals. Among the enrolled patients, only PFT data acquired within 3 months of the selected CT images were analyzed. For these patients, CPI and GAP stage were determined as previously described [20, 22]. The PFTs had been performed in accordance with the Japanese Respiratory Society guidelines [23].

**Fig 1:**
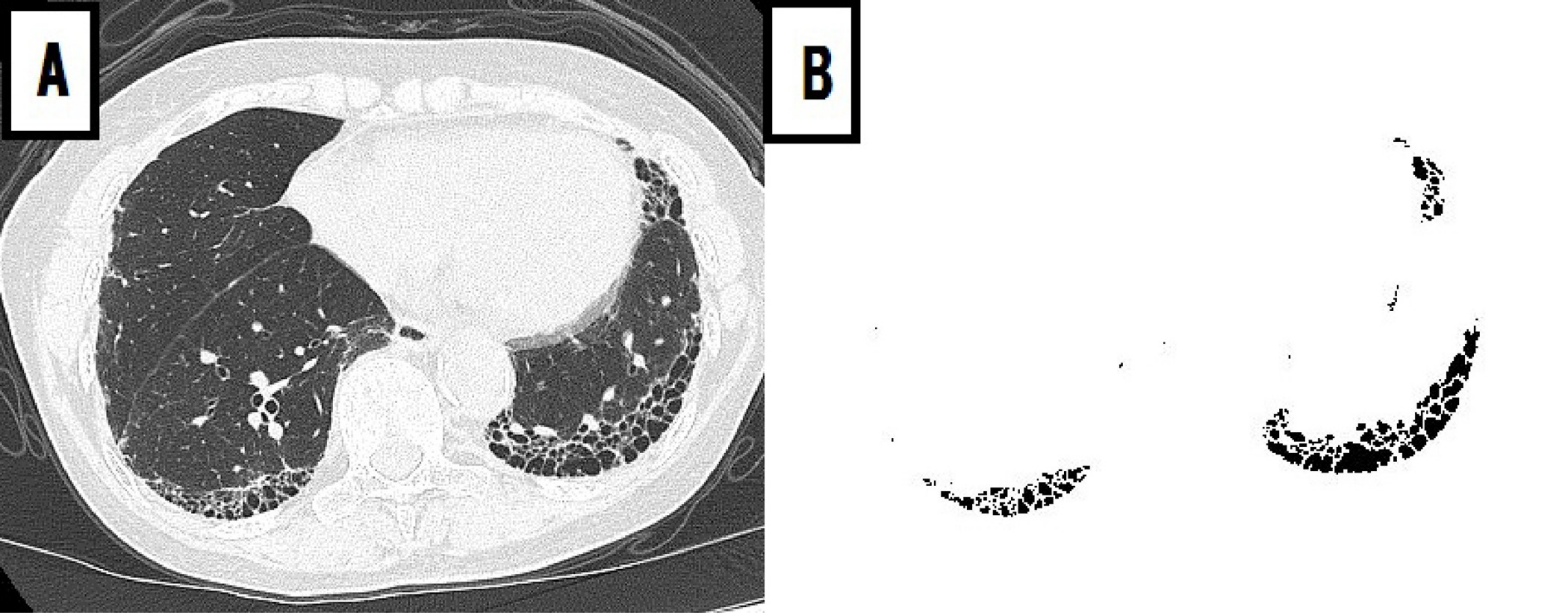
Honeycombing area detected by quantitative CT analysis. A 76-year-old man with idiopathic pulmonary fibrosis. (A) Original CT image was acquired at the level of the lower lungs using the lung window setting. (B) Honeycombing area was detected by quantitative CT analysis at the same level.

A computer-aided method for quantitative CT analysis of honeycombing area was used to automatically measure honeycombing areas on whole HRCT images, as previously described [19]. Briefly, quantitative CT analysis of honeycombing area involved the following process: (1) low-attenuation areas enclosed by thick walls were extracted as honeycombing areas; (2) the sum of honeycombing areas in all slices was calculated as the HA. The values of HA and percentage of HA to total lung area (%HA) were measured for each patient (Fig 1).

### Statistical analysis

Data are expressed as median and interquartile range (IQR). The correlation of HA with PFT findings and patient characteristics was evaluated by Spearman’s correlation coefficient analysis.

For assessment of 3-year mortality, the clinical features of alive and deceased patients were compared using the Mann–Whitney *U* test. The effect of each variable on 3-year mortality was evaluated by univariate logistic regression analysis adjusted by age, sex, body mass index (BMI), and pack-years. The model with the greatest predictive utility was determined by multivariable logistic regression analysis adjusted by age, sex, BMI, and pack-years using the stepwise method. The cutoff value for %HA was determined on the basis of the area under the receiver operating characteristic curve and Youden’s index [24]. Survival analysis was performed in accordance with the methods of Kaplan–Meier, with the endpoint being death. Differences in mortality were assessed by the log-rank test. All statistical analyses were performed using JMP version 9.0.2 (SAS Institute, Cary, NC). For all tests, *p* values < 0.05 were considered statistically significant.

## Results

### Patient characteristics

Of the 72 consecutive IPF patients with definite UIP pattern who visited the outpatient clinic of our hospital between April 2011 and March 2014, one was excluded for the lack of HRCT data at the first visit; additionally, 19 patients with follow-up period < 3 years were excluded. Thus, 52 patients with IPF were finally enrolled in this study. Among the 52 enrolled patients, 45 had corresponding PFT data acquired within 3 months of the selected CT images. The demographic, clinical, and physiologic characteristics of these 52 patients are summarized in Table 1.

**Table 1:** Demographic, clinical, and physiologic characteristics of patients with idiopathic pulmonary fibrosis.

**Characteristics**	**N**	**Value**
**Age, years**	52	75 (66–79)
**Male/Female**	52	44 (84.6%)/8 (15.4%)
**BMI, kg/m^2^**	52	22.7 (18.5–25.9)
**Smoking history (current or past/never)**	51	46 (90.2%)/5 (9.8%)
**Pack-years**	51	42.0 (19.5–61.5)
**FVC %pred., %**	45	83.6 (61.2–90.7)
**FEV_1_%pred., %**	45	80.2 (65.4–91.7)
**FEV_1_/FVC, %**	45	82.8 (76.1–86.8)
**DL_CO_ %pred., %**	37*	62.5 (51.5–76.7)
**CPI**	37	33.6 (27.2–42.2)
**KL-6, U/ml**	42	821 (626–1315)
**GAP stage (I/II/III)**	39	20/14/5
**Anti-fibrotic drug (use or non-use)**	52	6 (11.5%)/46 (88.5%)
**Follow-up period, years**	52	3.4 (1.2–4.2)

Data are presented as number or median and interquartile range.

BMI = body mass index; FVC = forced vital capacity; FEV_1_ = forced expiratory volume in 1 s; DL_CO_ = diffusing capacity of the lungs for carbon monoxide; CPI = composite physiologic index; KL-6 = Krebs von den Lungen-6; GAP = gender, age, and physiology.

*: two patients were not able to perform DL_CO._ Six patients did not have DL_CO_ data.

### CT quantitation of honeycombing area

The median (IQR) %HA was 3.8% (2.2–5.5%). The relationships between %HA and patient characteristics are shown in Table 2; %HA was significantly correlated with FVC, DL_CO_, and CPI (ρ, -0.53, *p* < 0.001; ρ, -0.46, *p* = 0.004; and ρ, 0.60, *p* < 0.001, respectively). Furthermore, %HA was significantly correlated with GAP stage (ρ, 0.51; *p* < 0.001).

**Table 2:** Relationship between patient characteristics and %HA determined by quantitative CT analysis.

	**%HA**	
	ρ	*p* value
**Age, years**	-0.16	0.267
**BMI, kg/m^2^**	-0.13	0.377
**Pack-years**	-0.09	0.515
**FVC %pred., %**	-0.53	< 0.001
**FEV_1_%pred., %**	-0.58	< 0.001
**DL_CO_ %pred., %**	-0.46	0.004
**CPI**	0.60	< 0.001
**KL-6, U/ml**	0.62	< 0.001
**GAP stage**	0.51	< 0.001

Data are presented as Spearman’s correlation coefficients. %HA = computed-tomography-derived %honeycombing area; BMI = body mass index; FVC = forced vital capacity; FEV_1_ = forced expiratory volume in 1 s; DL_CO_ = diffusing capacity of the lungs for carbon monoxide; CPI = composite physiologic index; KL-6 = Krebs von den Lungen-6; GAP = gender, age, and physiology.

### 3-year mortality and baseline parameters

Of the 52 patients, 23 were deceased and 29 alive at the end of the 3-year follow-up period. Deceased group had the lower BMI and %FVC (*p* = 0.028, and 0.035, respectively; Table 3) and the higher GAP stage and %HA (*p* = 0.005, and 0.004, respectively; Table 3). Table 4 presents the results of univariate logistic regression analysis of correlation between baseline variables and 3-year mortality. Among the evaluated clinical, physiologic, and radiographic characteristics, %HA, %FVC, CPI, and GAP stage were statistically significant predictors of mortality. Although %HA correlated with CPI and GAP stage (Table 2), multivariate logistic regression analysis revealed that only %HA (odds ratio [OR], 1.27; 95% confidence interval [CI], 1.05–1.62; *p* = 0.011) was a significant predictor of 3-year mortality.

**Table 3:** Comparison of clinical features between alive and deceased patients.

	Alive (n = 29)	Deceased (n = 23)	*p* value
**Age, years**	74 (66–77) (29)	76 (65–81) (23)	0.328
**Sex (male/female)**	25/4 (29)	19/4 (23)	0.722*
**BMI, kg/m**^**2**^	24.3 (20.8–26.2) (29)	21.6 (17.4–24.6) (23)	0.028
**Pack-years**	40.0 (19.1–64.5) (29)	42.0 (26.6–55.1) (22)	0.627
**FVC %pred., %**	84.4 (74.2–92.0) (26)	63.6 (53.5–88.7) (19)	0.035
**FEV**_**1**_**%pred., %**	73.4 (66.4–81.6) (26)	60.4 (54.1–66.9) (19)	0.113
**DL**_**CO**_ **%pred., %**	63.0 (52.3–77.3) (26)	62.5 (35.4–76.3) (11)	0.642
**CPI**	32.4 (26.6–40.1) (26)	37.6 (29.0–59.6) (11)	0.195
**KL-6, U/ml**	732 (636–959) (23)	904 (553–1810) (19)	0.404
**GAP stage**	1 (1–2) (26)	2 (1.5–3) (13)	0.005
**Anti-fibrotic drug (use/non-use)**	3/26 (29)	3/20 (23)	0.763*
**%HA, %**	3.2 (1.5–4.4) (29)	4.9 (3.4–8.0) (23)	0.004

Data are presented as number or median and interquartile range. *p* values derived by Mann–Whitney *U* test.

*p values derived by chi-square test.

%HA = computed-tomography-derived %honeycombing area; BMI = body mass index; FVC = forced vital capacity; FEV_1_ = forced expiratory volume in 1 s; DL_CO_ = diffusing capacity of the lungs for carbon monoxide; CPI = composite physiologic index; KL-6 = Krebs von den Lungen-6; GAP = gender, age, and physiology.

**Table 4:** Results of univariate logistic regression analysis for predictors of mortality.

	**OR**	**95% CI**	***p* value**
**%HA, %**	1.27	1.05–1.65	0.011
**FVC %pred., %**	0.95	0.91–0.99	0.009
**FEV_1_%pred., %**	0.97	0.93–1.00	0.076
**DL_CO_ %pred., %**	0.96	0.91–1.00	0.079
**CPI**	1.11	1.03–1.24	0.004
**KL-6, U/ml**	1.00	1.00–1.00	0.104
**GAP stage**	5.84	2.71–942.40	0.005

Data were derived by univariate logistic regression analysis adjusted by age, sex, BMI, and pack-years. OR = odds ratio; CI = confidence interval; %HA = computed-tomography-derived %honeycombing area; BMI = body mass index; FVC = forced vital capacity; FEV_1_ = forced expiratory volume in 1 s; DL_CO_ = diffusing capacity of the lungs for carbon monoxide; CPI = composite physiologic index; GAP = gender, age, and physiology; KL-6 = Krebs von den Lungen-6.

### Prediction of mortality by %HA

**Fig 2:**
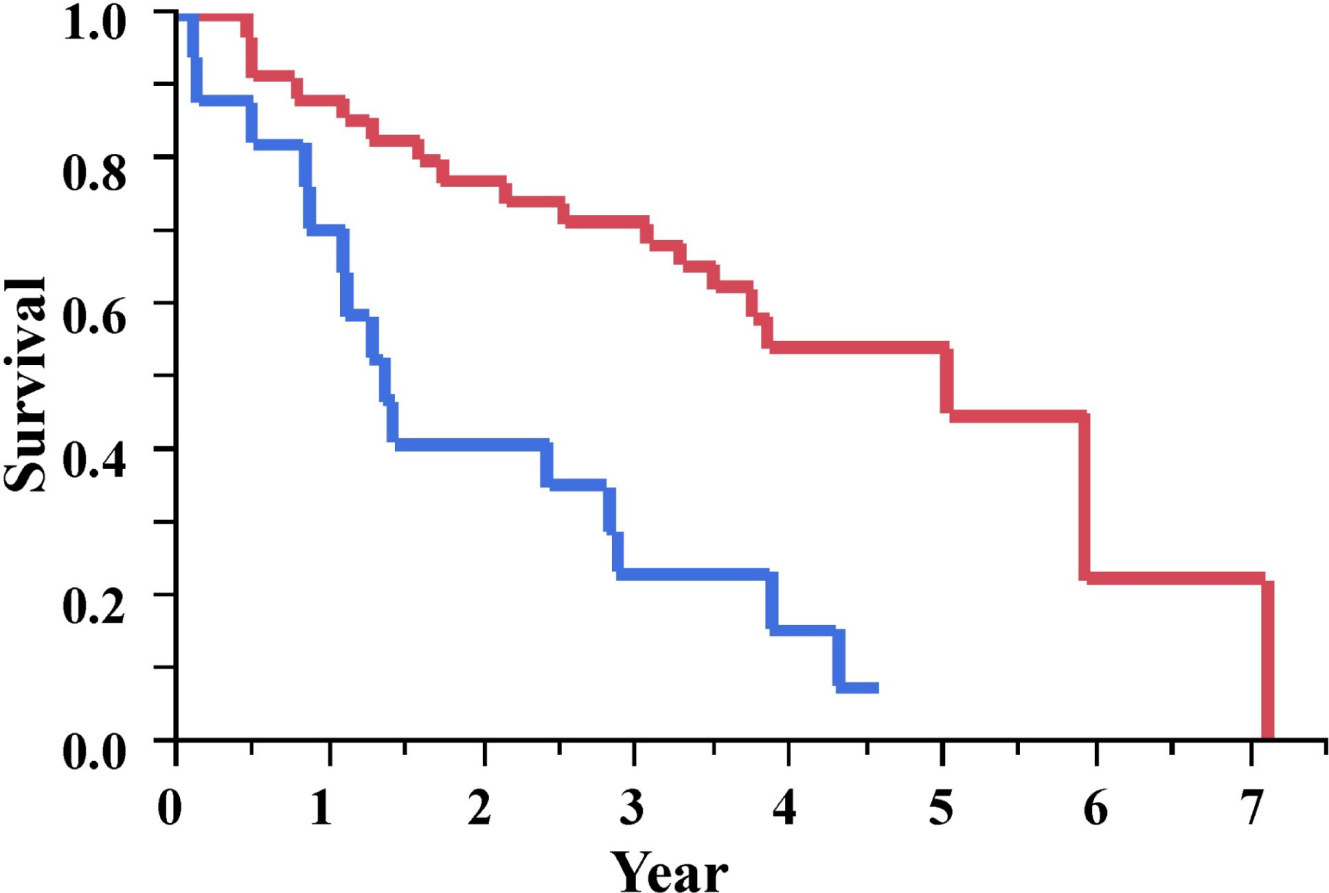
Kaplan–Meier plot of survival probability. The median survival times (interquartile ranges) were 1.3 (0.8–2.8) and 5.0 (2.1–5.9) years among patients with greater (≥ 4.8%; blue line) and smaller (< 4.8%; red line) %HA (log-rank test; p < 0.001), relative to the cutoff. %HA = computed-tomography-derived %honeycombing area.

From the results of receiver operating characteristic analysis, the Youden’s index and accuracy of %HA were estimated to be 4.8% and 73.1%, respectively (sensitivity, 86.2%; specificity, 56.5%; S1 Fig). The Kaplan–Meier survival curve demonstrated a significant difference in mortality, which were defined by a cutoff point of 4.8% for %HA (Fig 2). The median (IQR) survival times were 1.3 (0.8–2.8) and 5.0 (2.1–5.9) years, respectively, among patients with %HA greater and lesser than 4.8% (log-rank test; *p* < 0.001).

## Discussion

The 2011 international statement mentions that extent of honeycombing on HRCT images is an independent predictor of mortality in IPF, although there is no uniformity in approach to quantitation [4]. We hypothesized that honeycombing area measured by quantitative CT analysis might be associated with an increased risk of mortality in IPF. The present results demonstrated that %HA was significantly correlated with 3-year mortality (Table 4). Furthermore, %HA was an independent predictor of 3-year mortality as the result of multivariate analysis using the stepwise regression method. These findings show that %HA might be a quantitative prognostic factor in IPF.

Both FVC and DL_CO_ are considered the gold standards for assessment of IPF. However, it is important to consider that, in patients with IPF, FVC and DL_CO_ findings are confounded by coexisting emphysema [20, 21]. Emphysema is present in approximately one-third of patients with IPF [25]. FVC measurements may not be appropriate for monitoring disease progression in patients with IPF and severe emphysema [26]. In patients with IPF with coexistent emphysema, CPI is thought to be a more accurate prognostic determinant than any individual parameter of PFTs [20]. Additionally, in IPF, GAP stage is thought to be a better prognostic determinant than any individual parameter of PFTs [22, 27]. In the present study, %HA was significantly correlated with not only FVC and DL_CO_ but also CPI and GAP stage (Table 2). This is the retrospective cohort study of the consecutive IPF patients with HRCT scan data. Unfortunately, 7 patients did not have PFT within 3 months of the selected HRCT. It is inaccurate to discuss the relationship between selected HRCT and PFT performed at different time. Therefore, PFT of 7 patients was treated as missing data.

In order to identify better prognostic factors in IPF, we performed logistic regression analysis of correlation of 3-year mortality with %HA and other variables, including PFT findings. We selected the 3-year time point for mortality because several retrospective longitudinal studies have reported that the median survival time from the time of IPF diagnosis is between 2 and 3 years [4, 27–31]. Among the evaluated variables, only %HA was found to be significantly correlated with 3-year mortality upon multivariate logistic regression analysis using the stepwise method. This result showed that %HA was an independent and useful variable for prediction of 3-year mortality in the present study population. Among FVC, forced expiratory volume in 1 s (FEV_1_), DL_CO_, CPI, GAP stage, and %HA, all variables exhibited strong correlations with each other. We, therefore, performed bivariate logistic regression analysis for the relationship of FVC, FEV_1_, DL_CO_, CPI, and GAP stage with %HA (S1 Table) and found that %HA was a significant predictor of mortality in combination with each of the variables except CPI and GAP stage. On the other hand, in combination with each other, neither %HA nor CPI or GAP stage was a significant predictor of mortality. These results seem to be derived from the strong correlation between CPI and %HA, GAP stage and %HA. These results show that in addition to CPI and GAP stage, %HA was a useful variable for prediction of 3-year mortality.

In evaluating the mortality in IPF, the analysis for time to death is also helpful. In univariate Cox proportional hazards models, %HA, %FVC, %FEV_1_, and CPI were statistically significant predictors of mortality (S2 Table). In multivariate Cox proportional hazards models including all variables using the stepwise regression method, only %HA (hazard ratio [HR], 1.13; 95% CI, 1.04–1.21; *p* = 0.006) was significant predictor of mortality. The results of bivariate Cox proportional hazards models (S3 Table) were almost similar to those of bivariate logistic regression analysis.

Because we wanted to evaluate honeycombing area by quantitative CT analysis, the present study population only included patients with IPF with definite usual interstitial pneumonia pattern on HRCT images. Although our study population was not representative of the entire spectrum of IPF, the relationship between mortality and GAP stage in our population was almost similar to that reported in previous studies [22, 32] on IPF (S4 Table). In evaluation of prognosis, we believe that the present results might be comparable to the trend among the general IPF population.

According to the review of computer-based quantitative CT image analyses in IPF, there were 3 types of quantitative CT analyses; the density histogram analysis, the density mask analysis like our method, and texture classification analysis [33]. Including semi-quantitative analysis by visual scoring, each analysis has its merits that can be used in clinical settings. The texture classification analysis may be the most useful and reliable method for objective management of IPF. However, the specific CT index and the algorithm remain unclear [33]. It is desirable to establish a method which is transparent and which is freely available for widespread use, such as the low attenuation area (LAA) for chronic obstructive pulmonary disease (COPD) [34, 35].

The present method of quantitative CT analysis of percentage of honeycombing area has important advantages relative to previously reported methods. First, since this method does not require PFT data (especially DL_CO_), honeycombing area can be quantified in almost all cases of IPF. Second, this method does not involve interobserver or intraobserver errors and can be used by anybody as a public-domain computer program. Third, it allows quantitative assessment of prognosis in IPF. Our method using 10-mm interval images is not perfect. However, we think it is not inferior to the past methods using 3–4 CT images of fixed position such as aortic arch, carina, or right inferior pulmonary vein.

There are several limitations to the present study. First, this was a retrospective study with a small sample size, conducted at a single institution. Second, our patients did not receive uniform treatment for IPF. The rates of anti-fibrotic drugs usage were not different between the alive group and deceased group (Table 3). Third, we analyzed only IPF patients with definite UIP pattern to investigate whether the extent of honeycombing area was a prognostic predictor or not. In other words, we analyzed only the IPF patients with honeycombing area. We did not analyzed the patients without honeycombing area. A prospective multicenter study on quantitative analysis of honeycombing area is needed to confirm the usefulness of %HA in management of patients with IPF.

## Conclusions

Relative extension of honeycombing in comparison to lung volume measured by quantitative CT analysis might be an important and independent predictor of mortality in IPF patients with definite UIP pattern. Furthermore, this analysis might provide new insights into disease progression in the future.

## Supporting information

S1 Table Results of logistic regression analysis for predictors of mortality and relationship between %HA and other variables. (DOCX)

S2 Table Results of univariate Cox proportional hazards models for predictors of mortality. (DOCX)

S3 Table Results of Cox proportional hazards models for predictors of mortality and relationship between %HA and other variables. (DOCX)

S4 Table Comparison of predicted or observed mortality according to GAP stage. (DOCX)

S1 Fig The receiver operating characteristic curve with the relative Youden’s index. The area under the curve with 4.8% cutoff point of %HA was 0.735. The sensitivity, the specificity, and the accuracy were 86.2%, 56.5%, and 73.1%, respectively). %HA = computed-tomography-derived %honeycombing area.
(DOCX)
