# Correction: Quantitative CT analysis of honeycombing area predicts mortality in idiopathic pulmonary fibrosis with definite usual interstitial pneumonia pattern: A retrospective cohort study

**DOI:** 10.1371/journal.pone.0226214

**Published:** 2019-12-02

**Authors:** Hiroaki Nakagawa, Emiko Ogawa, Kentaro Fukunaga, Daisuke Kinose, Masafumi Yamaguchi, Taishi Nagao, Sachiko Tanaka-Mizuno, Yasutaka Nakano

S1 Fig, “The receiver operating characteristic curve with the relative Youdens’s index.” does not appear. Please view S1 Fig here.

## Supporting information

S1 Fig The receiver operating characteristic curve with the relative Youden’s index. The area under the curve with 4.8% cutoff point of %HA was 0.735. The sensitivity, the specificity, and the accuracy were 86.2%, 56.5%, and 73.1%, respectively. %HA = computed-tomography-derived %honeycombing area.
(TIF)
