# Supplementary material for: Quantitative CT analysis of honeycombing area predicts mortality in idiopathic pulmonary fibrosis with definite usual interstitial pneumonia pattern: A retrospective cohort study
Source: PLoS One. 2019 Mar 21;14(3):e0214278. doi: 10.1371/journal.pone.0214278 (PMC6428407; doi:10.1371/journal.pone.0214278)
Supplement: S1 Table — (DOCX) [file pone.0214278.s001.docx]

**S1 Table.** Results of logistic regression analysis for predictors of mortality and relationship between %HA and other variables

|  | OR | 95% CI | *p* value |
| --- | --- | --- | --- |
| %HA, % | 1.87 | 1.20–3.57 | 0.003 |
| FVC %pred., % | 0.99 | 0.94–1.04 | 0.639 |
| %HA, % | 2.10 | 1.31–4.23 | <0.001 |
| FEV_1_ %pred., % | 1.01 | 0.96–1.06 | 0.647 |
| %HA, % | 2.15 | 1.17–5.66 | 0.009 |
| DL_CO_ %pred., % | 1.01 | 0.94–1.10 | 0.773 |
| %HA, % | 1.64 | 0.87–3.96 | 0.134 |
| CPI | 1.05 | 0.93–1.20 | 0.403 |
| %HA, % | 1.42 | 0.90-2.67 | 0.142 |
| GAP stage | 3.56 | 0.80–20.45 | 0.096 |

Data were derived by bivariate logistic regression analysis adjusted by age, sex, BMI, and pack-years.

OR = odds ratio; CI = confidence interval; %HA = computed-tomography-derived %honeycombing area; FVC = forced vital capacity; FEV_1_ = forced expiratory volume in 1 s; DL_CO_ = diffusing capacity of the lungs for carbon monoxide; CPI = composite physiologic index; GAP = gender, age, and physiology.
