# Supplementary material for: Quantitative CT analysis of honeycombing area predicts mortality in idiopathic pulmonary fibrosis with definite usual interstitial pneumonia pattern: A retrospective cohort study
Source: PLoS One. 2019 Mar 21;14(3):e0214278. doi: 10.1371/journal.pone.0214278 (PMC6428407; doi:10.1371/journal.pone.0214278)
Supplement: S2 Table — (DOCX) [file pone.0214278.s002.docx]

**S2 Table.** Results of univariate Cox proportional hazards models for predictors of mortality

|  | HR | 95% CI | *p* value |
| --- | --- | --- | --- |
| %HA, % | 1.13 | 1.04–1.21 | 0.006 |
| FVC %pred., % | 0.96 | 0.94–0.98 | <0.001 |
| FEV_1_ %pred., % | 0.97 | 0.94–0.99 | 0.008 |
| DL_CO_ %pred., % | 0.98 | 0.95–1.00 | 0.084 |
| CPI | 1.07 | 1.02–1.13 | 0.003 |
| KL-6, U/ml | 1.00 | 1.00–1.00 | 0.088 |
| GAP stage | 2.84 | 1.49-5.50 | 0.002 |

Data were derived by univariate Cox proportional hazards models adjusted by age, sex, BMI, and pack-years.

HR = hazard ratio; CI = confidence interval; %HA = computed-tomography-derived %honeycombing area; BMI = body mass index; FVC = forced vital capacity; FEV_1_ = forced expiratory volume in 1 s; DL_CO_ = diffusing capacity of the lungs for carbon monoxide; CPI = composite physiologic index; KL-6 = Krebs von den Lungen-6; GAP = gender, age, and physiology.
