# Supplementary material for: Quantitative CT analysis of honeycombing area predicts mortality in idiopathic pulmonary fibrosis with definite usual interstitial pneumonia pattern: A retrospective cohort study
Source: PLoS One. 2019 Mar 21;14(3):e0214278. doi: 10.1371/journal.pone.0214278 (PMC6428407; doi:10.1371/journal.pone.0214278)
Supplement: S3 Table — (DOCX) [file pone.0214278.s003.docx]

**S3 Table.** Results of Cox proportional hazards models for predictors of mortality and relationship between %HA and other variables

|  | HR | 95% CI | *p* value |
| --- | --- | --- | --- |
| %HA, % | 1.23 | 1.02–1.46 | 0.028 |
| FVC %pred., % | 0.98 | 0.95–1.01 | 0.119 |
| %HA, % | 1.29 | 1.08–1.54 | 0.006 |
| FEV_1_ %pred., % | 0.99 | 0.96–1.02 | 0.549 |
| %HA, % | 1.48 | 1.09–2.06 | 0.012 |
| DL_CO_ %pred., % | 1.00 | 0.96–1.04 | 0.989 |
| %HA, % | 1.26 | 0.90–1.80 | 0.179 |
| CPI | 1.04 | 0.98–1.11 | 0.198 |
| %HA, % | 1.29 | 0.99–1.71 | 0.060 |
| GAP stage | 1.86 | 0.78–4.20 | 0.158 |

Data were derived by bivariate Cox proportional hazards models adjusted by age, sex, BMI, and pack-years.

HR = hazard ratio; CI = confidence interval; %HA = computed-tomography-derived %honeycombing area; FVC = forced vital capacity; FEV_1_ = forced expiratory volume in 1 s; DL_CO_ = diffusing capacity of the lungs for carbon monoxide; CPI = composite physiologic index; GAP = gender, age, and physiology.
