# Supplementary material for: Quantitative CT analysis of honeycombing area predicts mortality in idiopathic pulmonary fibrosis with definite usual interstitial pneumonia pattern: A retrospective cohort study
Source: PLoS One. 2019 Mar 21;14(3):e0214278. doi: 10.1371/journal.pone.0214278 (PMC6428407; doi:10.1371/journal.pone.0214278)
Supplement: S4 Table — (DOCX) [file pone.0214278.s004.docx]

**S4 Table.** Comparison of predicted or observed mortality according to GAP stage

|  |  | Stage I | | |  | Stage II | | |  | Stage III | | |
| --- | --- | --- | --- | --- | --- | --- | --- | --- | --- | --- | --- | --- |
| Mortality, % |  | 1-y | 2-y | 3-y |  | 1-y | 2-y | 3-y |  | 1-y | 2-y | 3-y |
| Present study |  | 0.0 | 10.0 | 15.0 |  | 21.4 | 21.4 | 28.6 |  | 20.0 | 80.0 | 80.0 |
| Ley B et al., 2012 [21] |  | 5.6 | 10.9 | 16.3 |  | 16.2 | 29.9 | 42.1 |  | 39.2 | 62.1 | 76.8 |
| Kim ES et al., 2015 [31] |  | 2.5 | 8.3 | 16.6 |  | 21.9 | 38.4 | 50.7 |  | 21.1 | 39.5 | 52.6 |

Data are presented as percentage.

Observed 1-, 2-, and 3-year mortality are presented according to GAP stage.

GAP = gender, age, and physiology.
