# Supplementary material for: Quantitative CT analysis of honeycombing area predicts mortality in idiopathic pulmonary fibrosis with definite usual interstitial pneumonia pattern: A retrospective cohort study
Source: PLoS One. 2019 Mar 21;14(3):e0214278. doi: 10.1371/journal.pone.0214278 (PMC6428407; doi:10.1371/journal.pone.0214278)
Supplement: S1 Fig — The area under the curve with 4.8% cutoff point of %HA was 0.735. The sensitivity, the specificity, and the accuracy were 86.2%, 56.5%, and 73.1%, respectively). %HA = computed-tomography-derived %honeycombing area. (DOCX) [file pone.0214278.s005.docx]

**Fig S1. The receiver operating characteristic curve with the relative Youden’s index.**

The area under the curve with 4.8% cutoff point of %HA was 0.735. The sensitivity, the specificity, and the accuracy were 86.2%, 56.5%, and 73.1%, respectively). %HA = computed-tomography-derived %honeycombing area.
