# Supplementary figures and images for: Correction: Quantitative CT analysis of honeycombing area predicts mortality in idiopathic pulmonary fibrosis with definite usual interstitial pneumonia pattern: A retrospective cohort study
Source: PLoS One. 2019 Dec 2;14(12):e0226214. doi: 10.1371/journal.pone.0226214 (PMC6886836; doi:10.1371/journal.pone.0226214)

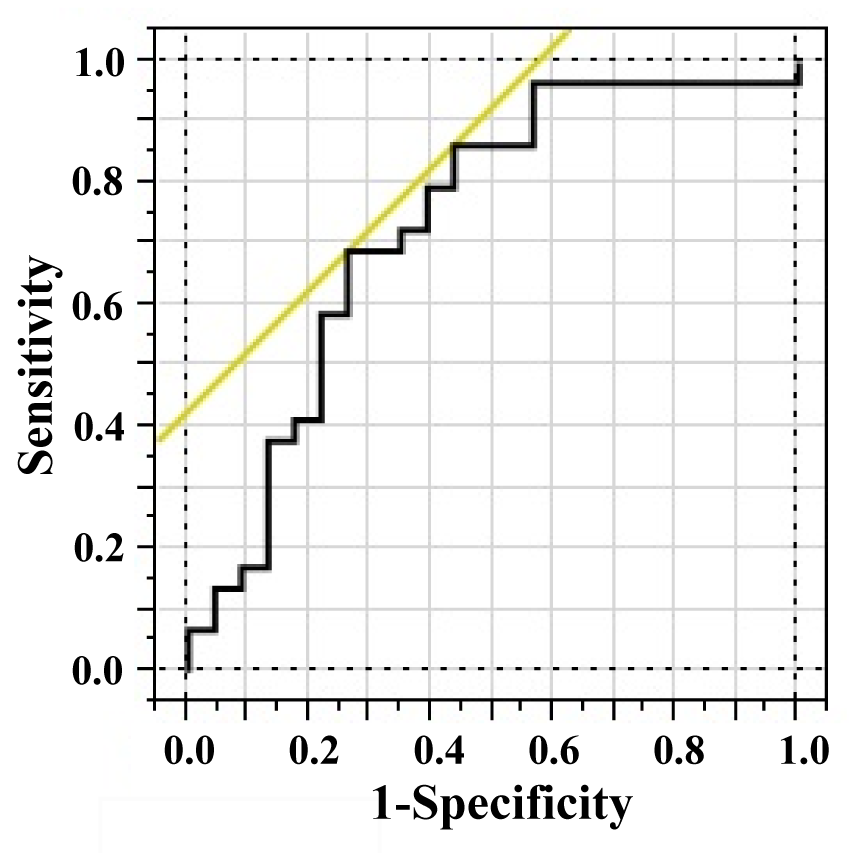

Supplement: S1 Fig — The area under the curve with 4.8% cutoff point of %HA was 0.735. The sensitivity, the specificity, and the accuracy were 86.2%, 56.5%, and 73.1%, respectively. %HA = computed-tomography-derived %honeycombing area. (TIF) [file pone.0226214.s001.tif]
